# The concordance between the evolutionary trend and the clinical manifestation of the two SARS-CoV-2 variants

**DOI:** 10.1093/nsr/nwab073

**Published:** 2021-05-28

**Authors:** Ben Hu, Ran Liu, Xiaolu Tang, Yuchen Pan, Ming Wang, Yongqing Tong, Guangming Ye, Gaigai Shen, Ruochen Ying, Aisi Fu, Di Li, Wanxu Zhao, Jing Peng, Jie Guo, Dong Men, Xinmin Yao, Yirong Wang, Hong Zhang, Zhihui Feng, Junping Yu, Liangjun Chen, Zixin Deng, Xuemei Lu, Ya-Ping Zhang, Yirong Li, Bende Liu, Lilei Yu, Yan Li, Jian Lu, Tiangang Liu

**Affiliations:** Key Laboratory of Combinatorial Biosynthesis and Drug Discovery, Ministry of Education and School of Pharmaceutical Sciences, Wuhan University, China; Key Laboratory of Combinatorial Biosynthesis and Drug Discovery, Ministry of Education and School of Pharmaceutical Sciences, Wuhan University, China; CAS Key Laboratory of Quantitative Engineering Biology, Shenzhen Institute of Synthetic Biology, Shenzhen Institute of Advanced Technology, Chinese Academy of Sciences, China; State Key Laboratory of Protein and Plant Gene Research, Center for Bioinformatics, School of Life Sciences, Peking University, China; Department of Cardiology, Renmin Hospital of Wuhan University, China; Department of Clinical Laboratory, Renmin Hospital of Wuhan University, China; Department of Clinical Laboratory, Renmin Hospital of Wuhan University, China; Department of Clinical Laboratory Medicine, Zhongnan Hospital of Wuhan University, China; Key Laboratory of Combinatorial Biosynthesis and Drug Discovery, Ministry of Education and School of Pharmaceutical Sciences, Wuhan University, China; State Key Laboratory of Protein and Plant Gene Research, Center for Bioinformatics, School of Life Sciences, Peking University, China; Key Laboratory of Combinatorial Biosynthesis and Drug Discovery, Ministry of Education and School of Pharmaceutical Sciences, Wuhan University, China; Department of Clinical Laboratory, Renmin Hospital of Wuhan University, China; Key Laboratory of Combinatorial Biosynthesis and Drug Discovery, Ministry of Education and School of Pharmaceutical Sciences, Wuhan University, China; Wuhan Dgensee Clinical Laboratory Co., Ltd., China; Wuhan Dgensee Clinical Laboratory Co., Ltd., China; CAS Key Laboratory of Special Pathogens and Biosafety, Centre for Biosafety Mega-Sciences, Wuhan Institute of Virology, Chinese Academy of Sciences, China; State Key Laboratory of Protein and Plant Gene Research, Center for Bioinformatics, School of Life Sciences, Peking University, China; State Key Laboratory of Protein and Plant Gene Research, Center for Bioinformatics, School of Life Sciences, Peking University, China; State Key Laboratory of Protein and Plant Gene Research, Center for Bioinformatics, School of Life Sciences, Peking University, China; Wuhan Dgensee Clinical Laboratory Co., Ltd., China; CAS Key Laboratory of Special Pathogens and Biosafety, Centre for Biosafety Mega-Sciences, Wuhan Institute of Virology, Chinese Academy of Sciences, China; Department of Clinical Laboratory Medicine, Zhongnan Hospital of Wuhan University, China; Key Laboratory of Combinatorial Biosynthesis and Drug Discovery, Ministry of Education and School of Pharmaceutical Sciences, Wuhan University, China; State Key Laboratory of Genetic Resources and Evolution, Kunming Institute of Zoology; Center for Excellence in Animal Evolution and Genetics, Chinese Academy of Sciences, China; State Key Laboratory of Genetic Resources and Evolution, Kunming Institute of Zoology; Center for Excellence in Animal Evolution and Genetics, Chinese Academy of Sciences, China; Department of Clinical Laboratory Medicine, Zhongnan Hospital of Wuhan University, China; Department of Emergency Medicine, First People's Hospital of Jiangxia, China; Department of Cardiology, Renmin Hospital of Wuhan University, China; Department of Clinical Laboratory, Renmin Hospital of Wuhan University, China; State Key Laboratory of Protein and Plant Gene Research, Center for Bioinformatics, School of Life Sciences, Peking University, China; Key Laboratory of Combinatorial Biosynthesis and Drug Discovery, Ministry of Education and School of Pharmaceutical Sciences, Wuhan University, China

**Keywords:** coronavirus, SARS-CoV-2, COVID-19, clinical severity, genetic variant

Coronavirus disease 2019 (COVID-19), the disease caused by infection with severe acute respiratory syndrome coronavirus 2 (SARS-CoV-2), has developed into a global pandemic that continues to pose an enormous threat to public health and the global economy. Since the first SARS-CoV-2 genome was released [1], thousands of genetic variants have been identified in SARS-CoV-2 strains isolated from worldwide patients. Some variants in the spike (S) protein have been reported to be associated with functional changes in viral transmissibility or viral loads, such as the D614G variant [2,3], the N501Y change, the P681H substitution and deletion of ΔH69-ΔV70 [4]. However, there is currently little direct evidence linking the genomic variants and clinical severity of COVID-19.

Based on the variants at two significantly linked SNP sites 8782 (in *orf1ab*) and 28 144 (in *ORF8*) (NC_045 512 as reference), SARS-CoV-2 genomes can be categorized into S lineage (U8782 and C28 144) or L lineage (C8782 and U28 144). The categorization was first proposed based on the analysis of 103 SARS-CoV-2 genomes in our previous study [5], subsequently confirmed by other studies [6–8]. Of note, the S and L lineage corresponds to the A and B lineage in Rambaut’s *et al*. A/B nomenclature system [8]. Among the 103 genomes, the L lineage was more prevalent than the S lineage (∼70% versus ∼28%), and evolutionary analysis inferred that S was ancestral, and L was the derived form [5,9]. As recently demonstrated [7], ∼99.8% of the SARS-CoV-2 strains sequenced from global samples during the pandemic can be categorized into either the L or S lineage, suggesting the delineation of L and S lineages is robust (see Supplementary data for the analysis of 127 119 SARS-CoV-2 genomes deposited in the Global Initiative on Sharing All Influenza Data (GISAID, https://www.gisaid.org; as of October 19, 2020)). Interestingly, the L lineage was more prevalent than S as the COVID-19 pandemic developed, and the S-genomes almost disappeared after the end of June 2020 (Fig. S1).

Did the patients with L- or S-lineage SARS-CoV-2 experience different clinical outcomes? To address this issue, we collected SARS-CoV-2 samples from 271 patients diagnosed with COVID-19 in the early outbreak of the pandemic from five Wuhan hospitals (see Patient data source section of Supplementary data, Table S1). These patients were randomly recruited with regard to the viral lineage. Their admission dates spanned two key time points: January 23, 2020, when Wuhan implemented the entire city lockdown, and February 14, 2020, when Wuhan adopted closed-off community management. Because the strict city lockdown was implemented rapidly, largely confining the spread of SARS-CoV-2 to a closed environment within Wuhan, these SARS-CoV-2 samples are well suited for investigating the difference in clinical features associated with the two SARS-CoV-2 lineages.

Among the 271 patients, SARS-CoV-2 in 73 (26.9%) cases were S lineage, and in 198 (73.1%) cases were L lineage (see Supplementary Methods, Workflow I, and Table S2 for details). Although many genetic variants arose in both the L and S lineages as the pandemic continued [7], we believe the L- and S-lineage viruses isolated from the 271 patients were very close to the reference genome (NC_045 512). The major differences between the two lineages surveyed here were located at sites 8782 and 28 144, and neither lineage carried the G614 variant in the S protein (see Supplementary data and Fig. S2 for details). We gathered clinical features from the 271 patients’ medical records for statistical analysis alongside the SARS-CoV-2 lineages. There was no significant difference in overall clinical outcomes (i.e. the proportion of patients that were recovered and discharged) between patients of the two lineages (Table S3). According to the *Guidelines on the Diagnosis and Treatment of Novel Coronavirus* issued by the National Health Commission, China (7th Edition), the 271 patients were divided into four groups of clinical severity: mild (n = 24), moderate (n = 98), severe (n = 117) and critical disease (n = 32). A significantly higher proportion of S-lineage patients were in the severe or critical condition relative to the L-lineage patients (*P* = 0.011, Fisher's exact test, Fig. 1A). Considering the relatively small number of patients in the mild (21 for L and 3 for S) and critical (18 for L and 14 for S) groups, we grouped the mild and moderate patients into a ‘non-serious’ category and severe and critical patients into a ‘serious’ category, as previously performed [10]. A significantly higher proportion of the S-lineage patients (69.9%, 51/73) fell into the serious category, relative to L-lineage patients (49.5%, 98/198) (*P* = 0.004, Fig. 1B).

**Figure 1.**
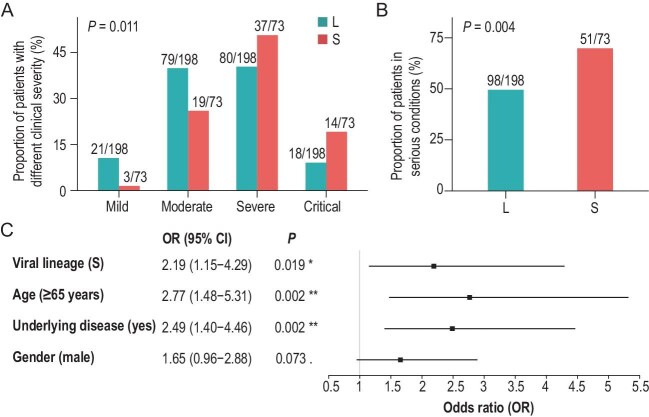
The L- and S-lineage SARS-CoV-2 are associated with different clinical severity of COVID-19. (A). Among all patients (n = 271), a significantly higher proportion of S-lineage patients (n = 73) was in a severe or critical condition, relative to L-lineage patients (n = 198). The number of L- or S-lineage patients that are in a category of clinical severity and the total number of patients with that SARS-CoV-2 lineage are shown as a fraction. (B). Among all patients (n = 271), a significantly higher proportion of the S-lineage patients was in serious condition than L-lineage patients. The number of L- or S-lineage patients in serious conditions and the total number of patients with that SARS-CoV-2 lineage are shown as fractions. (C). Results of the multivariate logistic regression of clinical severity against viral lineage, age, underlying medical condition and gender of the patients. Patients infected with the S-lineage SARS-CoV-2, the elderly (≥65 years), or those with underlying diseases tended to have a serious clinical severity. OR: odds ratio. The mean and 95% CI of OR are given in the right panel.

Confounding factors such as age, gender and underlying medical conditions affect the symptoms and clinical severity of COVID-19 [11]. Here, Fisher's exact tests were used to examine the relationships between clinical severity (non-serious versus serious) and age (<65 versus ≥65 years old), underlying medical conditions (without versus with underlying diseases) and gender (female versus male). The results showed that elderly (≥65 years) patients, males, and patients with underlying medical conditions were more likely to appear in the serious category (all *P* values were < 0.05, Fig. S3 and Table S4), which confirmed the previous conclusions. To further evaluate the potential influences of these confounding factors on patients’ outcomes, we performed a multivariate logistic regression analysis of clinical severity against the variables, including the previous three confounding factors and viral lineage (L versus S). As shown in Fig. 1C, in the multivariate

analysis, both age and underlying medical conditions of patients were significantly associated with clinical severity (*P* = 0.002 for both factors), but the effect of gender on clinical severity was marginal (*P* = 0.073), and the possible interaction between age and underlying medical conditions had a non-significant effect on clinical severity (*P* > 0.4). Intriguingly, we still detected a significant association between viral lineage and the clinical severity of patients in the multivariate regression analysis, with S-lineage patients more likely falling into the serious category (OR = 2.19, 95% CI 1.15–4.29, *P* = 0.019, Fig. 1C). This result was robust when we changed the cutoff for age stratification (<60 versus ≥60 years old; *P* = 0.009, Fig. S4A) or treating age as a continuous variable (*P* = 0.034, Fig. S4B) in the multivariate logistic regression analysis. Thus, after excluding potential confounding factors (age, gender and underlying medical conditions), we still found that patients with S-lineage SARS-CoV-2 had significantly greater odds of experiencing severe disease than patients with L-lineage SARS-CoV-2.

Of note, a previous study surveyed 112 COVID-19 patients in Shanghai, China, and detected no significant difference in clinical severity between the L- and S-lineage patients [10]. The inconsistency between that study and ours might be partially because the fraction of serious cases in Hubei Province (Wuhan is the capital city of Hubei) tended to be higher than that in other areas of China (27.50% versus 5.21%, Fig. S5). Also, the hospitals from which the vast majority of patients were recruited were the designated hospitals to treat serious (severe/critical) COVID-19 patients in Wuhan, which gave rise to a higher proportion of serious cases in this study (∼55%) than the previous one (∼19%). Thus, the larger sample size and the higher proportion of serious cases gave us more power in the statistical tests to detect the difference.

How the L- and S-lineages differ in patients’ clinical severity remains unknown. Zhang *et al*. reported the ORF8 protein (28 144 was in ORF8) could mediate immune evasion by downregulating MHC-I molecules, but both L- and S-lineage SARS-CoV-2 ORF8 showed a similar effect on down-regulating MHC-I [12]. Yao *et al*. demonstrated that among the patient-derived SARS-CoV-2 isolates, S lineage showed lower viral titer in Vero cells compared to L lineage SARS-CoV-2 isolates [13]. However, the detailed mechanisms deserve further studies.

In summary, here, we analyzed 271 COVID-19 patients in the early outbreak in Wuhan and detected a significant difference in clinical severity between the L- and S-lineage patients. Although it remains unclear when and where the L and S lineages split, our finding is consistent with the hypothesis that the pathogenicity of SARS-CoV-2 might have been attenuated during the evolution from the S to L lineage. One limitation of this study is that most patients recruited in this study were from the designated hospitals to treat serious COVID-19 patients in Wuhan. The asymptomatic patients were under-represented in this study because relatively fewer of them were hospitalized in the early stage of the pandemic. Moreover, a recent population-level study on anti-SARS-CoV-2 antibodies in Wuhan showed that most people positive for pan-immunoglobulins were asymptomatic [14]. Thus, more studies are required to deepen our understanding of the connections between clinical manifestations and genetic variants of SARS-CoV-2 during the pandemic.

## Supplementary Material

nwab073_Supplemental_File
