# Supplementary material for: The concordance between the evolutionary trend and the clinical manifestation of the two SARS-CoV-2 variants
Source: Natl Sci Rev. 2021 May 28;8(8):nwab073. doi: 10.1093/nsr/nwab073 (PMC8244779; doi:10.1093/nsr/nwab073)
Supplement: nwab073_Supplemental_File [file nwab073_supplemental_file.pdf]

Supplementary Information

1. Categorizing lineages of SARS-CoV-2 genomes from GISAID

We downloaded 138,931 SARS-CoV-2 genome sequences from Global Initiative on Sharing All Influenza Data (GISAID) (https://www.gisaid.org) [1] on October 19, 2020 (See **Table S5** for the acknowledgment list). Only viruses infecting human hosts were selected, and low-coverage sequences (> 5% Ns) and incomplete (< 29,000 nucleotides after removing Ns) genomes were excluded from downstream analysis. We used MAFFT v7.453 (parameter: --auto) [2] to align each genome to the reference sequence (Wuhan-Hu-1, GenBank: NC_045512, GISAID: EPI_ISL_402125). snp-sites (-v) [3] was used to identify SNVs and BCFtools v1.8 (merge --force-samples -O v) [4] was used to process the vcf files. Genomes with degenerate nucleotides or gaps at sites 8,782/28,144 and 3,037/14,408/23,403 except for genomes with gaps spanning the ~380 nucleotide deletion in *ORF8* (sites 27,848 to 28,229) [5, 6] were further excluded, resulting in a final set of 127,119 high-quality genomes and 252 of them were collected from Wuhan, China.

These SARS-CoV-2 genomes were then categorized into the "S lineage" (U8,782 and C28,144) or "L lineage" (C8,782 and U28,144) as previously described [7]. Among these genomes, 120,958 (95.15%) could be assigned to the L lineage (C8,782 and U28,144), 5,950 (4.68%) could be assigned to the S lineage (U8,782 and C28,144), and only 211 (0.17%) could not be categorized as the L or S lineage. These results suggest that the SNPs at sites 8,782 and 28,144, initially identified based on only 103 viral samples [7], are indeed very tightly linked, and SARS-CoV-2 can be categorized into the L or S lineage in 99.8% of strains that have been sequenced from global samples.

2. Constructing the phylogenetic tree

127,119 genomes were aligned to the reference genome using MAFFT. The 5' (sites 1-220) and 3' end (sites 29,675-29,903) relative to the reference genome were trimmed. The 10,556 high-quality genomes in GISAID prior to May 2, 2020, were used in the tree reconstruction. We used the genome sequences of bat coronavirus RaTG13 (GenBank accession number: MN996532) and GD Pangolin-CoV (merged from GISAID: EPI_ISL_410544 and Genome Warehouse: GWHABKW00000000) as outgroup as previously described [7]. We used RAxML v8.2.12 [8] to construct the phylogenetic tree with the maximum likelihood method. The phylogenetic tree was visualized with Interactive Tree Of Life (iTOL) v5 (https://itol.embl.de/).

The phylogenetic analysis well revealed the distinction between the L and S lineage (**Fig. S1A)**. As shown elsewhere [9], the L lineage could be further divided into L1 and L2 sublineage based on three tightly linked variants, with L1 carrying the ancestral allele (C3037, C14408, and A23403) and L2 carrying the derived allele (U3037, U14408, and G23403) (**Fig. S1A**). Of note, the A23403G mutation causes the D614G amino acid change in the S protein. Hence, the L1 sublineage, to which the reference genome (NC_045512) belonged, carried the ancestral D614 variant; and genomes in the L2 sublineage carried the derived G614 variant.

3. The relative prevalence of S and L lineage during the development of the pandemic

Among the 127,119 high-quality SARS-CoV-2 genomes available from GISAID, 124,233 had detailed information on the dates they were isolated. The number of genomes was summarized in two-week intervals, and the frequency of S, L1, and L2 clade, as well as the genomes which could not be categorized in either S or L lineage (Other), or L1 or L2 sublineage (L*) in each interval was calculated.

A salient observation is that L was more prevalent than S as the COVID-19 pandemic developed, and this pattern is especially pronounced for the L2 sublineage (the SARS-CoV-2 genomes were stratified based on the dates they were isolated, **Fig S1B**). For instance, the vast majority of viral genomes isolated after May 1, 2020, belonged to the L2 sublineage. In contrast, the relative prevalence of both L1- and S-genomes kept decreasing since the beginning of February 2020; and the S-genomes almost disappeared after the end of June 2020 (**Fig. S1B)**. Although these patterns might be somewhat affected by sampling bias of the sequenced genomes, these observations overall suggest that during the spreading of SARS-CoV-2, L tended to prevail over S; and within the L lineage, L2 was more prevalent than L1.

4. SARS-CoV-2 genomes from Wuhan tended to be very close to the reference genome

There were 252 high-quality SARS-CoV-2 genomes in GISAID (as of October 19, 2020) that were sampled from Wuhan, China (virus isolation dates ranging from December 24, 2019 to March 29, 2020). Among these genomes, 38 (15.1%) belonged to S, and 191 (75.8%) belonged to L. Interestingly, all the 191 L-genomes belonged to L1, and none of them belonged to L2 (i.e., none of them carried the G614 variant in the S protein). Compared to the reference genome (NC_045512), viruses isolated later tended to carry more variants (**Fig. S2A**). Accordingly, the 252 genomes from Wuhan tended to have substantially fewer variants than the remaining samples in GISAID (mean ± sd is 2.4 ± 2.5 and 9.4 ± 4.3 in CDSs for the former and latter, respectively). The Fst analysis (**Fig. S2B**) of the genomes from Wuhan revealed no other sites substantially differentiated between these two lineages except sites 8,782 and 28,144.

Supplementary Methods

1. Patient data source

A total of 271 patients diagnosed with COVID-19 during the period from January 9 to May 8, 2020, were collected from 5 hospitals in Wuhan, China (**Workflow I**). All COVID-19 infections were confirmed by the diagnostic criteria in the "New Coronavirus Pneumonia Diagnosis and Treatment Program" issued by the National Health Commission of the People's Republic of China. The infection with SARS-CoV-2 for each patient was confirmed positive by real-time reverse transcription-polymerase chain reaction (RT-qPCR) detection at two positions (*orf1ab* and *N* gene). The same samples used for patient diagnosis were used to sequence viral genomes and analyzed in this study.

The patients' admission dates spanned January 23, 2020, when Wuhan as a whole was placed under quarantine (city blockage), and February 14, 2020, when Wuhan implemented lockdown within the community (community blockage). Both time points are important in the study. Patients were treated at five hospitals, including the main campus of the Renmin Hospital of Wuhan University (RHWU) (38 patients); the First People's Hospital of Jiangxia District (9 patients), mainly receiving outpatients; the east campus of RHWU (170 patients), which mainly received patients in serious condition; Zhongnan Hospital of Wuhan University, which mainly received outpatients and non-serious patients (45 patients), and a mobile cabin hospital (9 patients), which mainly received non-serious patients (**Table S1 & S2**).

2. Clinical data collection

This study and the usage of medical records were approved by the Ethics Committees of RHWU (WDRY2020-K061), including the main campus of RHWU, the eastern campus of RHWU, and the mobile cabin hospital; the Ethics Committees of Zhongnan Hostpital of Wuhan University (2020066); and the Ethics Committees of First People's Hospital of Jiangxia District (2020024). The requirement for informed consent was waived by the Ethics Committees. All medical record data were collected by a team of professional clinicians. The clinicians collected clinical information from the electronic medical records in the hospital information systems (HIS) of the five hospitals, and the information was classified according to SARS-CoV-2 lineages (**Table S3**). After exporting patients' existing medical records from the HIS, we developed a standardized data collection form (SDCF) by extracting key information, such as demographics, treatments received, and clinical outcomes. These data were independently reviewed by two researchers to ensure the accuracy of data collection. If key information could not be obtained from the electronic medical records, the researchers collected it through

communication with the attending doctors and other medical staff; if the data was still missing, the field was recorded as "unknown". The SDCF was regularly verified and updated in two-week intervals until each case had a final clinical outcome, i.e., until discharge or until a death record was issued. At last, 9 of 271 patients lacked age and gender information and 250 patients had complete information including underlying medical condition.

The epidemiological assessment in this study included disease complications and clinical severity. The complications during hospitalization were defined according to the Guidelines on the Diagnosis and Treatment of Novel Coronavirus issued by the National Health Commission, China (7th Edition) [10], and the WHO Interim Guidance Document about the novel coronavirus (https://www.who.int/docs/default-source/coronaviruse/clinical-management-of-novel-cov.pdf). The clinical severity was categorized into four stages by professional clinicians, i.e., mild, moderate, severe, and critical, according to the Guidelines on the Diagnosis and Treatment of Novel Coronavirus issued by the National Health Commission, China (7th Edition). Briefly, mild cases were defined as mild clinical symptoms and no pulmonary radiological manifestations. Moderate cases showed fever, respiratory symptoms, and radiological manifestations of pneumonia. Severe cases were defined by meeting one of the following criteria: respiratory distress > 30 breaths/min; oxygen saturation levels (SpO2) ≤ 93% at rest; arterial partial pressure of oxygen (PaO2)/fraction of inspired oxygen (FiO2) (PaO2/FiO2 ratio) ≤ 300 mmHg; or chest imaging showing obvious lesion progression exceeding 50% within 24-48 hours. Critical cases were defined by meeting one of the following criteria: respiratory failure and mechanical ventilation requirement; shock; or with other organ failures that require ICU care. The clinical severity was also categorized into two groups: non-serious cases (including mild/moderate cases) and serious cases (including severe/critical cases).

The demographic assessment mainly included gender, age, clinical symptoms (symptoms at admission), and underlying medical conditions. The clinical symptoms were those considered by the US Centers for Disease Control and Prevention (CDC) Symptoms of Coronavirus (https://www.cdc.gov/coronavirus/2019-ncov/symptoms-testing/symptoms.html) or as reported in previous reports [11-14] as the symptoms of COVID-19, including fever, chill, cough, fatigue, sore throat, shortness of breath, dyspnea, myalgia, arthralgia, dizziness, headache, poor appetite, diarrhea, and nausea or vomiting. The underlying medical conditions included diabetes, hypertension, cardiovascular and cerebrovascular disease, respiratory system disease, malignancy/organ transplantation, and others according to the International Classification of Diseases, 10th Edition [15].

The therapy plan evaluation mainly included oxygen therapy, antibiotic treatment, antifungal treatment, glucocorticoids, and immune enhancers (following Guidelines on the Diagnosis and Treatment of Novel Coronavirus, 7th Edition). The clinical outcome assessment included the patient's duration of hospitalization, improvement and discharge, transfer to another hospital, or death. The transferred patients were still included in this study, but only the information on their first admission was recorded, not the information after transfer.

3. Statistical analysis

All statistical analyses were performed by a joint team of clinicians and statisticians. All categorical variables were described as frequency rates and percentages. The continuous variable "age" was converted to a categorical variable by dividing patients into two groups: adult (< 65) and the elderly (≥ 65 years) unless explicitly stated otherwise. Fisher's exact test was conducted to determine whether the two lineages differed in clinical features. We performed a multivariate logistic regression analysis of clinical severity ("non-serious" as 0, and "serious" as 1) against the variables including viral lineage (L versus S), patient age (< 65 versus ≥ 65 years old), underlying medical conditions (without versus with underlying disease), and gender (female versus male). Analyses were performed using R (www.r-project.org). A two-sided α of less than 0.05 in the final multivariate model was considered statistically significant.

4. Reverse transcription of viral RNA

The RNA samples that the viral lineages were categorized by Sanger sequencing or NTS were reverse transcripted into cDNA. Extracted RNA from each specimen was subjected to the reverse transcription procedure using the PrimeScript II 1st strand cDNA synthesis kit (Takara Bio, 6210A). The reverse transcription reaction mixture, preparation protocol, and thermal cycling settings were determined according to the manufacturer's instructions. Amplification was performed in a C1000 thermal cycler (Bio-Rad, USA) using the following conditions: 5 min at 65°C followed by quickly cooling down on ice for RNA degeneration, then 10 min at 30°C, 30 min at 42°C, 5 min at 95°C, and cooling on ice. The PCR products were stored at -40°C.

5. Determination of SARS-CoV-2 lineage by Sanger sequencing

Prior to Sanger sequencing, two polymerase chain reaction (PCR) reactions were used to amplify the appropriate regions covering sites 8,782 and 28,144, respectively. A series of specific primer pairs (**Table S6**) was designed using National Center for Biotechnology Information (NCBI) online Primer-BLAST (https://www.ncbi.nlm.nih.c/tools/primer-blast/)

tool. For samples in which target regions were amplified by one round of PCR, the master mix consisted of 15 μL of KOD One PCR Master Mix (TOYOBO, KMM-101), to which 1 μL of each of the forward and reverse primer (final concentration 0.3 μM), 2 μL of products of reverse transcription, and nuclease-free water (up to a total volume of 30 μL) were added. The conditions of the one-round PCR were as follows: 1 cycle at 98°C for 3 min and 30 cycles at 95°C for 15 s, 55°C for 5 s, and 68°C for 5 s, followed by a final elongation step at 68°C for 5 min. For samples in which target regions were amplified by two-round nested PCR reaction, the first-round reaction was identical to the one-round PCR excluding the primer pair, and 5 μL products and nuclease-free water were added to a total volume of 30 μL, whereupon the annealing temperature of the second round reaction was increased to 60°C. Negative controls were set across the whole PCR process, and the products of samples and negative controls were validated by agarose gel electrophoresis. If the negative wells of the electrophoretogram were clean, the target band of the sample was purified for Sanger sequencing. If the base at site 8,782 exhibited T and/or 28,144 exhibited C, the SARS-CoV-2 strain was categorized as S lineage; if the base at site 8,782 exhibited C and/or 28,144 only exhibited T, the SARS-CoV-2 strain was determined to be L lineage.

6. Categorization of SARS-CoV-2 lineage by NTS

We recently developed nanopore targeted sequencing (NTS) [16], a method for the accurate and comprehensive detection of SARS-CoV-2. This method was based on the amplification of 11 virulence-related fragments and one specific gene fragment of SARS-CoV-2, using a primer panel developed in-house. The amplified regions covered site 28,144 but not site 8,782 on the SARS-CoV-2 genome. The primer for the target region covering site 28,144 is listed in **Table S7**. The amplification procedure was performed under the conditions described in our previous study [16]. The sequencing libraries were constructed using the 1D Ligation Kit (SQK-LSK109; Oxford Nanopore, UK) and sequenced using Oxford Nanopore GridION. In addition, MinKNOW (v. 3.6.5) and Porechop (v. 0.2.4) (https://github.com/rrwick/Porechop) were used for base-calling and barcode demultiplexing of sequencing data, respectively. High quality reads (Q score > 7, classified into "pass" by MinKNOW) [17-19] were retained to be mapped to human cDNA (GRCh38) and SARS-CoV-2 reference genome (NC_045512) using Minimap2 (v2.17) [20]. Reads aligned to human cDNA were discarded. We used SAMtools (v1.3.1) [4] mpileup to identify the SNVs at sites that had enough sequencing depth (≥ 10) with reads having MAPQs ≥ 20. For site 28,144, the sequencing depth cutoff was 50. If the base at site 28,144 only exhibited C, the SARS-CoV-2 strain was categorized as S lineage; if the base at site 28,144 only exhibited T, the SARS-CoV-2 strain was categorized as L lineage.

7. Categorizing SARS-CoV-2 lineage by in-house RT-qPCR

The 20 μL reaction contained 2 μL of RNA, 5 μL of RT-qPCR Reaction Mix buffer provided with the LightCycler Multiplex RNA Virus Master (Roche, Mannheim, Germany), 0.1 μL of RT enzyme solution from the kit, 4.05 μL of primer Mix, and 8.85 μL of Water (PCR Grade). Primer Mix (**Table S6**) has been optimized for categorizing L and S lineages of SARS-CoV-2 (**Figure S6**). All oligonucleotides were synthesized and provided by Sangon Biotech (Shanghai, China). Thermal cycling was performed at 50°C for 10 min for reverse transcription, followed by 95°C for 30 s and then 45 cycles of 95°C for 5 s and 60°C for 30 s. We used BioRad CFX96 instruments (BioRad, Hercules, USA) to perform RT-qPCR.

The performance of in-house RT-qPCR in categorizing SARS-CoV-2 lineage was validated by Sanger sequencing. Among the 27 samples used in the cross-validation, all of them showed the same result in RT-qPCR and Sanger sequencing (**Table S8**), indicating the in-house RT-qPCR method can accurately identify the S and L lineages of SARS-CoV-2.

REFERENCE

1. Shu, Y. and J. McCauley, *GISAID: Global initiative on sharing all influenza data - from vision to reality.* Euro Surveill, 2017. **22**(13).
2. Katoh, K. and D.M. Standley, *MAFFT multiple sequence alignment software version 7: improvements in performance and usability.* Mol Biol Evol, 2013. **30**(4): p. 772-80.
3. Page, A.J., et al., *SNP-sites: rapid efficient extraction of SNPs from multi-FASTA alignments.* Microb Genom, 2016. **2**(4): p. e000056.
4. Li, H., et al., *The Sequence Alignment/Map format and SAMtools.* Bioinformatics, 2009. **25**(16): p. 2078-9.
5. Gong, Y.-N., et al., *SARS-CoV-2 genomic surveillance in Taiwan revealed novel ORF8-deletion mutant and clade possibly associated with infections in Middle East.* Emerging Microbes & Infections, 2020. **9**(1): p. 1457-1466.
6. Su, Y.C.F., et al., *Discovery and Genomic Characterization of a 382-Nucleotide Deletion in ORF7b and ORF8 during the Early Evolution of SARS-CoV-2.* mBio, 2020. **11**(4): p. e01610-20.
7. Tang, X., et al., *On the origin and continuing evolution of SARS-CoV-2.* National Science Review, 2020. **7**(6): p. 1012-1023.
8. Stamatakis, A., *RAxML version 8: a tool for phylogenetic analysis and post-analysis of large phylogenies.* Bioinformatics, 2014. **30**(9): p. 1312-3.
9. Tang, X., et al., *Evolutionary analysis and lineage designation of SARS-CoV-2 genomes.* Science Bulletin, 2021. DOI: 10.1016/j.scib.2021.02.012
10. National Health Commission of the People's Republic of China. *Diagnosis and Treatment Protocol for COVID-19 (Trial Version 7)*. 2020; Available from: http://en.nhc.gov.cn/2020-03/29/c_78469.htm.
11. Huang, C., et al., *Clinical features of patients infected with 2019 novel coronavirus in Wuhan, China.* The Lancet, 2020. **395**(10223): p. 497-506.
12. Wang, D., et al., *Clinical Characteristics of 138 Hospitalized Patients With 2019 Novel Coronavirus–Infected Pneumonia in Wuhan, China.* JAMA, 2020. **323**(11): p. 1061-1069.
13. Pan, F., et al., *Time Course of Lung Changes a Chest CT during Recovery from Coronavirus Disease 2019 (COVID-19).* Radiology, 2020. **295**(3): p. 715-721.
14. Shi, H., et al., *Radiological findings from 81 patients with COVID-19 pneumonia in Wuhan, China: a descriptive study.* The Lancet Infectious Diseases, 2020. **20**(4): p. 425-434.
15. World Health Organization. *ICD-10: international statistical classification of diseases and related health problems: tenth revision (2004 edition)*. 2004.
16. Wang, M., et al., *Nanopore Targeted Sequencing for the Accurate and Comprehensive Detection of SARS-CoV-2 and Other Respiratory Viruses.* Small, 2020: p. 2002169.
17. Charalampous, T., et al., *Nanopore metagenomics enables rapid clinical diagnosis of bacterial lower respiratory infection.* Nat Biotechnol, 2019. **37**(7): p. 783-792.
18. Leggett, R.M., et al., *Rapid MinION profiling of preterm microbiota and antimicrobial-resistant pathogens.* Nat Microbiol, 2020. **5**(3): p. 430-442.
19. Jain, M., et al., *Improved data analysis for the MinION nanopore sequencer.* Nat Methods, 2015. **12**(4): p. 351-6.

20. Li, H., *Minimap2: pairwise alignment for nucleotide sequences.* Bioinformatics, 2018. **34**(18): p. 3094-3100.

Table S1. The samples were collected from five different hospitals

**Hospital**	**Date of admission**	**Patient intake**	**Typed—no. (%)**
First People's Hospital of Jiangxia District	2020.01.19-2020.01.27	Outpatient	9 (3.3)
Mobile cabin hospital†	2020.02.03-2020.03.10	Mild and regular patients	9 (3.3)
Main Campus of RHWU‡	2020.01.16-2020.05.08	Outpatients	38 (14.0)
East Campus of RHWU* ‡	2020.01.17-2020.03.25	Outpatients, severe and critical patient	170 (62.7)
Zhongnan Hospital of Wuhan University	2020.01.09-2020.03.04	Outpatients, mild and regular patients	45 (16.6)
Total	2020.01.09-2020.05.08	All patients	271 (100.0)

‡ RHWU, Remin Hospital of Wuhan University

* This hospital was designated for severe and critical COVID-19 patients on January 30, 2020

† This hospital was designated for mild and regular COVID-19 patients on February 3, 2020

Table S2. Lineages of COVID-19 patients at 5 hospitals in Wuhan from January to May, 2020

Date of admission	Hospital 1 ‡		Hospital 2 ‡		Hospital 3 ‡		Hospital 4 ‡		Hospital 5 ‡		Cumulative count
	S	L	S	L	S	L	S	L	S	L	
Jan. 11										L (1)	S (0), L (1)
Jan. 13										L (1)	S (0), L (2)
Jan. 15				L (1)							S (0), L (3)
Jan. 17	S (1)	L (1)								L (1)	S (1), L (5)
Jan. 19						L (4)					S (1), L (9)
Jan. 21						L (3)				L (1)	S (1), L (13)
Jan. 23 ★						L (1)				L (1)	S (1), L (15)
Jan. 25	S (1)									L (2)	S (2), L (17)
Jan. 27						L (1)			S (2)	L (1)	S (4), L (19)
Jan. 29	S (2)	L (3)									S (6), L (22)
Jan. 31	S (8)	L (10)	S (2)	L (4)						L (1)	S (16), L (37)
Feb. 2	S (1)	L (7)					S (2)*	L (7)*		L (8)	S (19), L (59)
Feb. 4	S (7)	L (5)	S (1)						S (1)	L (3)	S (28), L (67)
Feb. 6	S (14)	L (43)	S (2)	L (2)						L (5)	S (44), L (117)
Feb. 8	S (8)	L (13)	S (2)	L (1)							S (54), L (131)
Feb. 10	S (4)	L (8)		L (1)							S (58), L (140)
Feb. 12		L (5)								L (1)	S (58), L (146)
Feb. 14 ★★		L (5)									S (58), L (151)
Feb. 16	S (1)	L (3)								L (1)	S (59), L (155)
Feb. 22	S (1)	L (3)	S (1)								S (61), L (158)
Feb. 24				L (1)						L (1)	S (61), L (160)
Feb. 28	S (1)	L (2)	S (1)							L (12)	S (63), L (174)
Mar. 3	S (1)	L (2)		L (1)					S (1)	L (1)	S (65), L (178)
Mar. 7	S (1)	L (3)	S (2)	L (1)							S (68), L (182)
Mar. 9	S (2)	L (2)		L (1)							S (70), L (185)
Mar. 21				L (1)							S (70), L (186)
Mar. 23			S (1)								S (71), L (186)
Mar. 25	S (1)	L (1)									S (72), L (187)
Apr. 8				L (2)							S (72), L (189)
Apr. 15			S (1)	L (4)							S (73), L (193)
Apr. 30				L (4)							S (73), L (197)
May 8				L (1)							S (73), L (198)
**Total**	**S (54)**	**L (116)**	**S (13)**	**L (25)**	**S (0)**	**L (9)**	**S (2)**	**L (7)**	**S (4)**	**L (41)**	**S (73), L (198)**

‡ Hospital 1: The East Campus of Renmin Hospital of Wuhan University
Hospital 2: The Main Campus of Renmin Hospital of Wuhan University
Hospital 3: The First People's Hospital of Jiangxia District
Hospital 4: The mobile cabin hospital
Hospital 5: Zhongnan Hospital of Wuhan University

* 9 cases from the mobile cabin hospital could not be traced back and were counted as Feb. 3, the date the hospital started to treat patients

★ Jan. 23, City blockade measures; ★★ Feb. 14, Community blockade measures

S lineage: green; L lineage: yellow

**Table S3. SARS-CoV-2 lineage correlation with clinical characteristics**						
**Characteristics**	**All patients (n = 271)**			**Group II (n = 101) €**		
	S lineage (n = 73)	L lineage (n = 198)	P value	S lineage (n = 26)	L lineage (n = 75)	*P* value
The lineage of SARS-CoV-2—no. (n, %)	73 (271, 26.9)	198 (271, 73.1)	-	26 (101, 25.7)	75 (101, 74.3)	-
Hospital※—no. (n, %)						
Hospital 1	54 (73, 74.0)	116 (198, 58.6)	0.003	20 (26, 76.9)	41 (75, 54.7)	0.079
Hospital 2	13 (73, 17.8)	25 (198, 12.6)		4 (26, 15.4)	9 (75, 12.0)	
Hospital 3	0 (73, 0)	9 (198, 4.5)		0 (26, 0)	6 (75, 8.0)	
Hospital 4	2 (73, 2.7)	7 (198, 3.5)		0 (26, 0)	0 (75, 0)	
Hospital 5	4 (73, 5.5)	41 (198, 20.7)		2 (26, 7.7)	19 (75, 25.3)	
Clinical outcome—no. (n, %)						
Recovered and discharged	64 (73, 87.7)	181 (198, 91.4)	0.358	26 (26, 100.0)	74 (75, 98.7)	1.000
Died	9 (73, 12.3)	17 (198, 8.6)		0 (26, 0)	1 (75, 1.3)	
Gender—no. (n, %)						
Female	35 (71, 49.3)	92 (191, 48.2)	0.890	13 (26, 50.0)	34 (75, 45.3)	0.820
Male	36 (71, 50.7)	99 (191, 51.8)		13 (26, 50.0)	41 (75, 54.7)	
Age—no. (n, %)						
20-64	37 (71, 52.1)	134 (191, 70.2)	0.008	26 (26, 100.0)	75 (75, 100.0)	1.000
65-96	34 (71, 47.9)	57 (191, 29.8)		0 (26, 0)	0 (75, 0)	
Underlying diseases—no. (n, %)						
Diabetes	8 (70, 11.4)	26 (180, 14.4)	0.682	0 (26, 0)	0 (75, 0)	1.000
Hypertension	20 (70, 28.6)	56 (180, 31.1)	0.761	0 (26, 0)	0 (75, 0)	1.000
Cardiovascular and cerebrovascular disease	12 (70, 17.1)	22 (180, 12.2)	0.310	0 (26, 0)	0 (75, 0)	1.000
Respiratory system disease	3 (70, 4.3)	13 (180, 7.2)	0.567	0 (26, 0)	0 (75, 0)	1.000
Malignancy/organ transplantation	3 (70, 4.3)	9 (180, 5.0)	1.000	0 (26, 0)	0 (75, 0)	1.000
Others	13 (70, 18.6)	16 (180, 8.9)	0.046	0 (26, 0)	0 (75, 0)	1.000
Complication—no. (n, %)						
Acute respiratory distress syndrome	3 (70, 4.3)	8 (180, 4.4)	1.000	0 (26, 0)	2 (75, 2.7)	1.000
Respiratory failure	12 (70, 17.1)	24 (180, 13.3)	0.430	1 (26, 3.8)	2 (75, 2.7)	1.000
Acute respiratory injury	1 (70, 1.4)	0 (180, 0)	0.280	0 (26, 0)	0 (75, 0)	1.000
Acute cardiac injury	0 (70, 0)	4 (180, 2.2)	0.579	0 (26, 0)	0 (75, 0)	1.000

Acute renal injury	0 (70, 0)	1 (180, 0.6)	1.000	0 (26, 0)	0 (75, 0)	1.000
Secondary infection	12 (70, 17.1)	27 (180, 15.0)	0.700	4 (26, 15.4)	9 (75, 12.0)	0.736
Multiple organ dysfunction syndrome	3 (70, 4.3)	4 (180, 2.2)	0.404	0 (26, 0)	0 (75, 0)	1.000
Others	3 (70, 4.3)	4 (180, 2.2)	0.404	1 (26, 3.8)	1 (75, 1.3)	0.451
Therapy—no. (n, %)						
Oxygen therapy—no. (n, %)						
No oxygen therapy	8 (70, 11.4)	53 (180, 29.4)	0.013	6 (26, 23.1)	36 (75, 48.0)	0.010
Low-flow nasal cannula	43 (70, 61.4)	91 (180, 50.6)		20 (26, 76.9)	31 (75, 41.3)	
Non-invasive ventilation or high-flow nasal cannula	12 (70, 17.1)	27 (180, 15.0)		0 (26, 0)	7 (75, 9.3)	
Invasive mechanical ventilation	7 (70, 10.0)	9 (180, 5.0)		0 (26, 0)	1 (75, 1.3)	
Antibiotic treatment‡—no. (n, %)‡						
Basic antibiotics	46 (70, 65.7)	114 (180, 63.3)	0.771	15 (26, 57.7)	39 (75, 52.0)	0.655
Upgrade antibiotics	10 (70, 14.3)	22 (180, 12.2)	0.676	1 (26, 3.8)	6 (75, 8.0)	0.674
Other antibiotics	14 (70, 20.0)	29 (180, 16.1)	0.461	6 (26, 23.1)	16 (75, 21.3)	1.000
Antifungal treatment—no. (n, %)	6 (70, 8.6)	9 (180, 5.0)	0.372	0 (26, 0)	2 (75, 2.7)	1.000
Glucocorticoids treatment—no. (n, %)	29 (60, 48.3)	72 (159, 45.3)	0.762	8 (21, 38.1)	27 (66, 40.9)	1.000
Intravenous immunoglobulin therapy—no. (n, %)	50 (70, 71.4)	94 (180, 52.2)	0.007	16 (26, 61.5)	37 (75, 49.3)	0.363
Clinical severity§—no. (n, %)						
Mild	3 (73, 4.1)	21 (198, 10.6)	0.011	0 (26, 0)	2 (75, 2.7)	0.074
Moderate	19 (73, 26.0)	79 (198, 39.9)		10 (26, 38.5)	46 (75, 61.3)	
Severe	37 (73, 50.7)	80 (198, 40.4)		16 (26, 61.5)	25 (75, 33.3)	
Critical	14 (73, 19.2)	18 (198, 9.1)		0 (26, 0)	2 (75, 2.7)	
Serious	51 (73, 69.9)	98 (198, 49.5)	0.004	16 (26, 61.5)	27 (75, 36.0)	0.037
Non-serious	22 (73, 30.1)	100 (198, 50.5)		10 (26, 38.5)	48 (75, 64.0)	
Clinical symptoms—no. (n, %)						
Fever or chill	52 (71, 73.2)	135 (191, 70.7)	0.759	18 (26, 69.2)	56 (75, 74.7)	0.613
Cough	42 (71, 59.2)	98 (191, 51.3)	0.269	18 (26, 69.2)	43 (75, 57.3)	0.355
Fatigue	22 (71, 31.0)	53 (191, 27.7)	0.646	9 (26, 34.6)	19 (75, 25.3)	0.447
Sore throat	3 (71, 4.2)	13 (191, 6.8)	0.569	3 (26, 11.5)	7 (75, 9.3)	0.715
Shortness of breath	21 (71, 29.6)	42 (191, 22.0)	0.255	9 (26, 34.6)	10 (75, 13.3)	0.038
Dyspnea	4 (71, 5.6)	23 (191, 12.0)	0.171	1 (26, 3.8)	5 (75, 6.7)	1.000
Myalgia or arthralgia	7 (71, 9.9)	22 (191, 11.5)	0.827	3 (26, 11.5)	11 (75, 14.7)	1.000

Dizziness or headache	4 (71, 5.6)	13 (191, 6.8)	1.000	2 (26, 7.7)	6 (75, 8.0)	1.000
Poor appetite	8 (71, 11.3)	24 (191, 12.6)	1.000	3 (26, 11.5)	7 (75, 9.3)	0.715
Diarrhea	6 (71, 8.5)	16 (191, 8.4)	1.000	3 (26, 11.5)	5 (75, 6.7)	0.421
Nausea or vomiting	2 (71, 2.8)	11 (191, 5.8)	0.524	2 (26, 7.7)	0 (75, 0.0)	0.064

※ Hospital 1: The East Campus of Renmin Hospital of Wuhan University;
Hospital 2: The Main Campus of Renmin Hospital of Wuhan University;
Hospital 3: The First People's Hospital of Jiangxia District;
Hospital 4: The mobile cabin hospital;
Hospital 5: Zhongnan Hospital of Wuhan University

€ Group II: < 65 years old and without underlying diseases patients (**information of Group II patients was provided for reference and was not used in the statistical tests**).

* *P* value < 0.05, statistically significant

‡ Antibiotic treatment:
Basic antibiotic treatment: Quinolones, Cephalosporins, Beta lactams, Azithromycin
Upgrade antibiotic treatment: Carbapenems, Vancomycin, Linezolid, Teicoplanin, Tigecycline, Polymyxin
Other antibiotic treatment: other antibiotics or unknown drugs

§ Clinical severity:
Serious: severe/critical
Non-serious: mild/moderate

Table S4. The correlation of clinical severity with age, gender and underlying diseases among those patients with detailed information on age (n = 262), gender (n = 262) and underlying medical condition (n = 250)

**Age—no.**	**20-64 (n = 171)**	**65-96 (n = 91)**	***P* value†**
Clinical severity—no. (n, %)			
Serious	78 (171, 45.6)	71 (91, 78.0)	**4.73E-07**
Non-serious	93 (171, 54.4)	20 (91, 22.0)	
**Gender—no.**	**female (n = 127)**	**male (n = 135)**	***P* value†**
Clinical severity—no. (n, %)			
Serious	64 (127, 50.4)	85 (135, 63.0)	**0.0461**
Non-serious	63 (127, 49.6)	50 (135, 37.0)	
**Underlying diseases—no.**	**no (n = 125)**	**yes (n = 125)**	***P* value†**
Clinical severity—no. (n, %)			
Serious	58 (125, 46.4)	91 (125, 72.8)	**3.315E-05**
Non-serious	67 (125, 53.6)	34 (125, 27.2)	

† *P* value was calculated by Fisher's exact probability method.

Table S5. Please refer to the PDF file for the list of specific acknowledgments of the authors, originating and submitting laboratories of the sequences from GISAID (https://www.gisaid.org/).

Table S6. Sequences of primer pairs used in NTS and Sanger sequencing and sequences of primers and probes for in-house RT-qPCR			
**Primer application**	**Location**	**Sequence (5'-3')**	**Amplicon size (bp)**
NTS	28,144	F: AGTTACGTGCCAGATCAGT<br>R: ATGTTGAGTGAGAGCGGTGA	810
One-round PCR	8,782	P1_F: TGACATGTGCAACTACTAGACAA<br>P1_R: GCAGCCAAAACACAAGCTGA	526
		P2_F: ACAAAGATAGCACTTAAGGGTGGT<br>P2_R: GGTACTGGCTTACCAGAAGCA	527
	28,144	P1_F: CTTGTCACGCCTAAACGAACA<br>P1_R: TGAGAGCGGTGAACCAAGAC	569
Nested PCR	8,782	NP1_F: ATTGATGGTGGTGTCACTCG<br>NP1_R: GGTACTGGCTTACCAGAAGCA	359
		NP2_F: TTGATGGTGGTGTCACTCGT<br>NP2_R: TCAGCAGCCAAAACACAAGC	319
		NP-H1_F: AGGCTATTGATGGTGGTGTCA<br>NP-H1_R: TGCAGCAATCAATGGGCAAG	140
	28,144	NP1_F: GAATTGTGCGTGGATGAGGC<br>NP1_R: TGAGAGCGGTGAACCAAGAC	375
		NP2_F: TGAATTGTGCGTGGATGAGG<br>NP2_R: ACTGCGTTCTCCATTCTGGT	306
		NP-H1_F: CGTGGATGAGGCTGGTTCT<br>NP-H1_R: GGGGTGCATTTCGCTGATTT	238
In-house RT-qPCR	28,144	F: CCAATTTAGGTTCCTGGCAATT<br>R: CACCCATTCAGTACATCGATATCG<br>Probe_PT: VIC- ATTGTAAAAGGTAAACAGG-MGB<br>Probe_PC: ROX-ATTGTAAAAGGTGAACAGG-MGB	81

Table S7. PCR primers used for 136 samples			
**Sequencing method**	**PCR Primer**	**Case ID**	**Count**
NTS	28,144<br>F/R	100, 104, 149, 173, 174, 183, 187, 189, 300, 303, 304, B03, C01, C12, D11, D12, E01, E11, F1004, F1005, F1014, F1064, F11, G12	24
Sanger	8,782<br>P1_F/R	315	1
	8,782<br>P2_F/R	75, 79, 82, 83, 95, 105, 122,123, 135, 144, 153, 162, 181, 246, 247, 249, 251, 255, 258, 266, 290, 292, 295, 297, 305, 306, 307, 309, 311, 312, 317, 323, 324, 329, 332, 344, 355, 394, 402, 414, 438, 455, 456, 460, 473, 539, 649, 656, 696, 704, 720, 726, 730, 733, 735, 741, 764, 765, 767, 770, 772, F1132	62
	8,782<br>NP1_F/R (1st round)<br>NP2_F/R (2nd round)	326, 377, 426, 457, 467, 470, 494, 525, 532, 549, 568, 570, 962, 969, 257	15
	8,782<br>NP1_F/R (1st round)<br>NP-H1_F/R (2nd round)	279, 376, 387, 452, 461, 462, 474, 475, 491, 502, 959	11
	28,144<br>P1_F/R	76, 82, 83, 95, 135, 153, 162, 181, 246, 247, 249, 258, 266, 277, 290, 292, 295, 297, 299, 305, 306, 307, 309, 310, 311, 312, 317, 323, 324, 329, 332, 355, 394, 402, 506, 514, 764, 765, 767, 770, 772, 811	42
	28,144<br>NP1_F/R (1st round)<br>NP2_F/R (2nd round)	257, 270, 315, 326, 426, 457, 470, 494, 525, 549, 570, 983, 993, 998, 1001	15
	28,144<br>NP1_F/R (1st round)<br>NP-H1_F/R (2nd round)	429, 507, 582, 969, 971, 980, 985, 995, 999, 1005, 1009, 1011	12

Table S8. The validation of RT-qPCR performance in categorizing SARS-CoV-2 different lineages			
**Case ID**	**Categorizing by Sanger sequencing**	**Sanger sequencing position**	**Categorizing by RT-qPCR**
123	L	Sanger, 8782	L
135	S	Sanger, 8782, 28144	S
247	L	Sanger, 8782, 28144	L
255	S	Sanger, 8782	S
258	L	Sanger, 8782, 28144	L
266	L	Sanger, 8782, 28144	L
277	S	Sanger, 28144	S
297	S	Sanger, 8782, 28144	S
306	S	Sanger, 8782, 28144	S
307	L	Sanger, 8782, 28144	L
317	L	Sanger, 8782, 28144	L
323	L	Sanger, 8782, 28144	L
332	S	Sanger, 8782, 28144	S
455	S	Sanger, 8782	S
456	L	Sanger, 8782	L
460	S	Sanger, 8782	S
473	L	Sanger, 8782	L
656	S	Sanger, 8782	S
720	S	Sanger, 8782	S
733	L	Sanger, 8782	L
741	L	Sanger, 8782	L
772	S	Sanger, 8782, 28144	S
811	L	Sanger, 28144	L
980	L	Sanger, 28144	L
983	L	Sanger, 28144	L
985	L	Sanger, 28144	L
998	S	Sanger, 28144	S

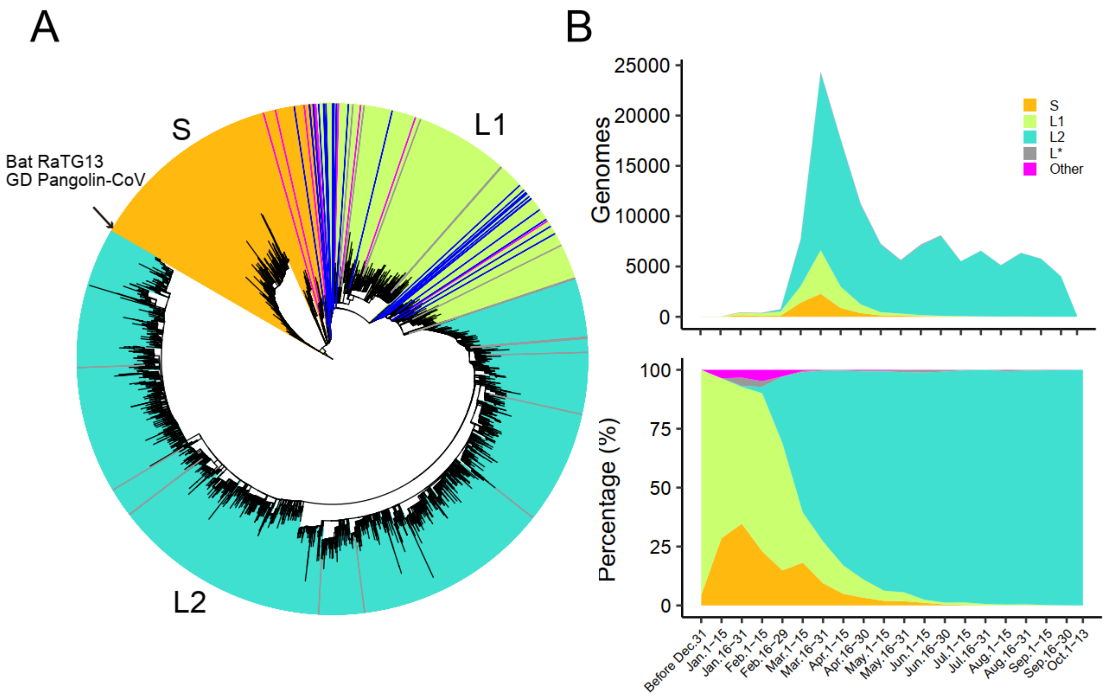

Figure S1. The phylogenetic tree and temporal distributions of the L1-, L2- and S-clade of SARS-CoV-2. Genomes from each clade are colored (S: orange; L1: light green; L2: mint). The genomes that could not be assigned to S or L (Other) were in magenta, and those that could not be assigned to L1 or L2 (L*) were in gray.

A). The phylogenetic tree of SARS-CoV-2 genomes. The 10,556 high-quality genomes in GISAID prior to May 2, 2020, were used in the tree reconstruction, and the tree was rooted with the bat coronavirus RaTG13 and GD Pangolin-CoV (coronavirus isolated from pangolins smuggled into Guangdong province in China). Among the 10,556 genomes, 8,896 (84.3%) belonged to the L lineage, 1,630 (15.4%) belonged to the S lineage, and only 30 (0.3%) could not be categorized as either L or S lineage. Note that S was clearly delineated from L, and L1 was clearly delineated from L2. Genomes collected in Wuhan (colored in navy) during the early outbreak all belonged to the S or L1 clade.

B). The worldwide numbers (upper) and proportions (lower) of S-, L1-, and L2-clade genomes among 124,223 high-quality genomes with detailed sampling date in GISAID (as of October 19, 2020) that were summarized at a two-week interval.

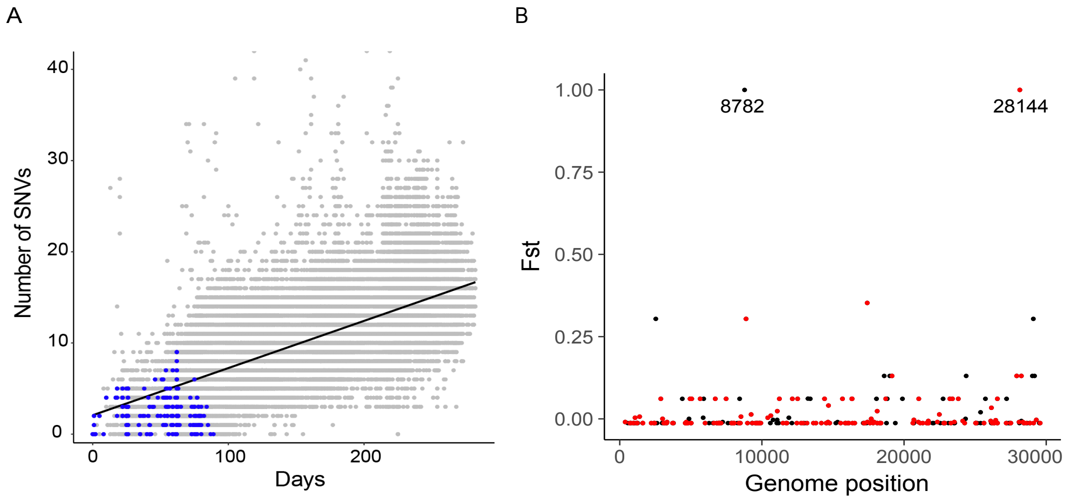

Figure S2. The relationship between the number of SNVs and collection date and the Fst values at SNV sites in the CDSs.

A). A positive correlation was observed between the number of SNVs identified in the CDS regions of one genome and the collection date. The *x*-axis represents the collection date of each genome relative to that of the reference genome (NC_045512, collected on December 31, 2019). The *y*-axis represents the number of SNVs identified in the CDS regions of each genome. Of note, 22 genomes collected earlier than December 31, 2019 were not considered in the regression analysis. Samples collected from Wuhan are colored in blue, and the others are colored in gray.

B). The calculated Fst value on each SNV site in the CDSs. The *x*-axis represents the position of each site relative to the reference genome. The *y*-axis represents the calculated Fst value. Red: sites with nonsynonymous variants; black: sites with other variants. The Fst values of SNV sites identified in 252 genomes from Wuhan in GISAID are displayed. Of note, only sites 8,782 and 28,144 that were used to delineate the L and S lineage had high Fst values, suggesting no other sites were likely to contribute to the difference observed between the L- and S-lineage in this study.

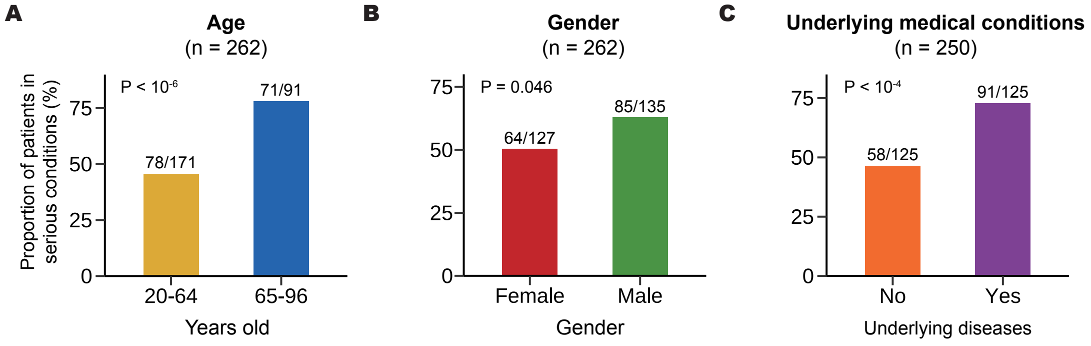

Figure S3. Risk factors affecting the clinical severity of COVID-19.
A). A significantly higher proportion of elderly patients (65–96 years) was in the serious category compared to adult patients (20–64 years).
B). A significantly higher proportion of male patients was in the serious category compared to female patients.
C). A significantly higher proportion of the patients with underlying diseases was in the serious category compared to the patients without underlying diseases.
In each panel, the y-axis represents the percentage (%) of patients in the serious category (defined as severe or critical cases, in contrast to the non-serious category, which included mild and moderate cases). In each group, the number of patients in the serious category and the total number of patients in that group are shown as a fraction.

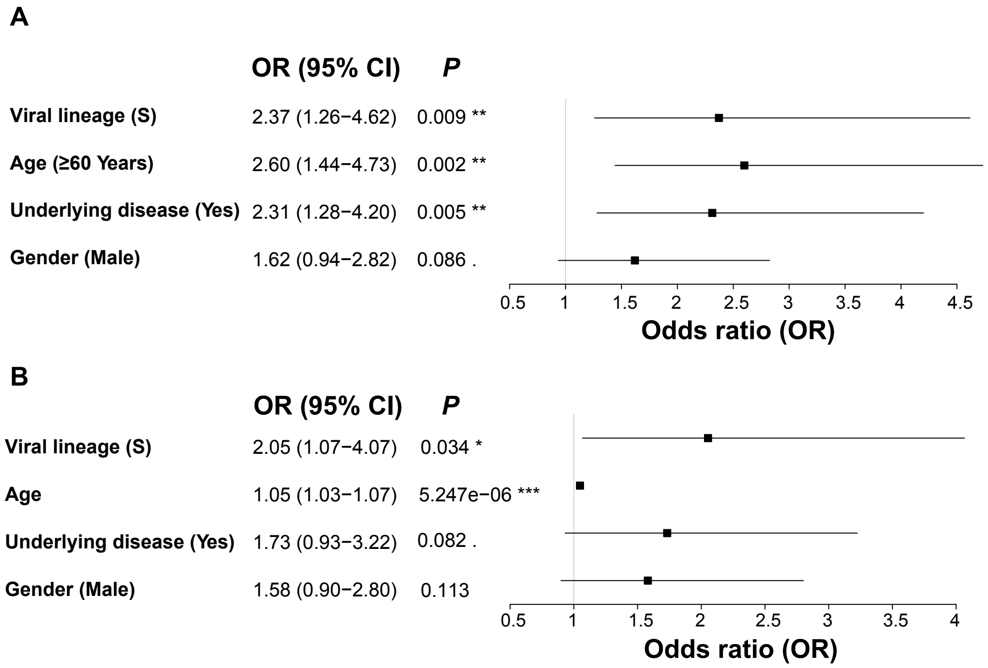

Figure S4. Results of the multivariate logistic regression of clinical severity against viral lineage, age, underlying medical conditions, and gender of the patients.
A). Age was divided into two groups (< 60 versus ≥ 60 years old).
B). Age was treatd as a continuous variable.
OR: odds ratio. The mean and 95% CI of OR are given in the right panel.

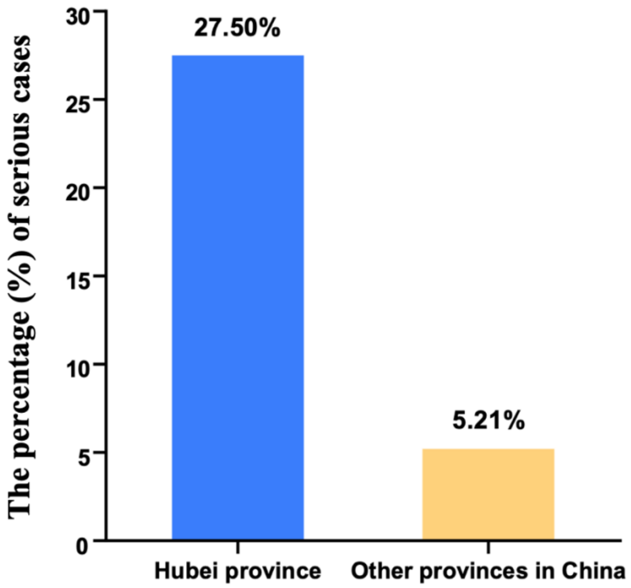

Figure S5. The percentage (%) of serious (severe/critical) cases in Hubei province and other provinces of China. The overall percentage of serious cases for Hubei province was calculated based on the average of daily percentage of serious cases from January 11, 2020 to April 23, 2020. The overall percentage of serious cases for other provinces in China was calculated based on the average of daily percentage of serious cases from February 5, 2020 to April 23, 2020 because data from January 11, 2020 to February 4, 2020 was not published on the official website. The calculations were based on data collected from the official website of Health Commission of Hubei Province (http://wjw.hubei.gov.cn/) and National Health Commission of the People's Republic of China (http://www.nhc.gov.cn/), respectively.

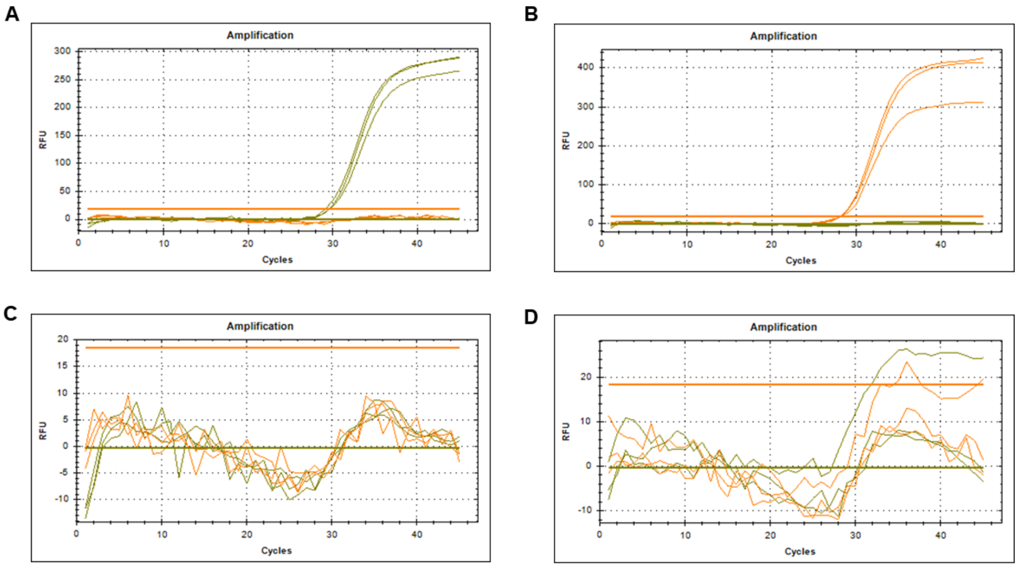

Figure S6. Specificity test of in-house RT-qPCR primers.
Two plasmids containing ORF8 mutants were constructed to simulate the two different lineages of SARS-CoV-2, including (A) pUC57-ORF8 (T28,144) and (B) pUC57-ORF8-C (C28,144), representing the L- and S-lineage SARS-CoV-2, respectively. In theory, the base at site 28,144 of SARS-CoV-2 may be A or G. Therefore, another two plasmids were constructed to simulate the hypothetical lineages, including (C) pUC57-ORF8-A (A28,144) and (D) pUC57-ORF8-G (G28,144). Primers were designed for RT-qPCR detection of L-lineage SARS-CoV-2 (T28,144) with VIC fluorescence signal (green color) and for detecting S-lineage SARS-CoV-2 with ROX fluorescence signal (orange color). Each RT-qPCR test was performed with the corresponding plasmids at 1,000,000 copies/mL using the in-house RT-qPCR method described in the method section and repeated three times (n = 3). The results showed that this RT-qPCR reaction could efficiently categorize L- or S-lineage SARS-CoV-2 because only VIC fluorescence signal was detected in pUC57-ORF8, and only ROX fluorescence signal was detected in pUC57-ORF8-C.

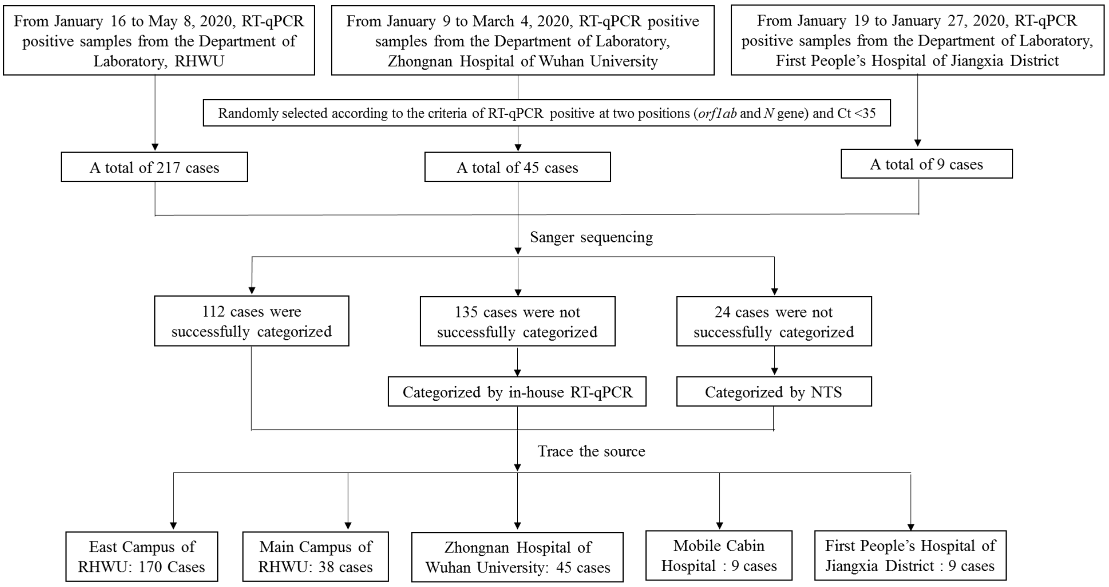

Workflow I. The process of sample collection. The Laboratory Department of RHWU collected all the samples from the two campuses of RHWU and a small number of the samples from the mobile cabin hospital.
